# Characteristic Analysis of Occupational Confined Space Accidents in Taiwan and Its Prevention Strategy

**DOI:** 10.3390/ijerph17051752

**Published:** 2020-03-07

**Authors:** Chien-Chen Chiu, Yi-Ming Chang, Terng-Jou Wan

**Affiliations:** 1 Graduate School of Engineering Science and Technology, National Yunlin University of Science and Technology, No. 123 University Road, Section 3, Douliou, Yunlin 64002, Taiwan; 2 Commission for General Education, Center for General Education, National United University, No. 2, Lienda, Miaoli 36063, Taiwan; a0919666178@gmail.com; 3 Center for General Education, National Formosa University, No. 64, Wunhua Road, Huwei, Yunlin 63201, Taiwan; 4 Department of Safety Health and Environmental Engineering, National Yunlin University of Science and Technology, No. 123 University Road, Section 3, Douliou, Yunlin 64002, Taiwan

**Keywords:** confined space, systematic causal analysis technique (SCAT), medium, non-standard acts, non-standard conditions

## Abstract

According to the US Bureau of Labor Statistics (BLS), 882 people were killed or injured due to confined space accidents in 2011–2017. Occupational accident statistics published in 2008–2018 by the Taiwan Occupational Safety and Health Administration (OSHA, Taiwan) show that 70 people suffered from disasters and 52 were injured in the 64 accident reports involving confined spaces. In the US, on average, 126 people die each year in accidents related to confined spaces, and in Taiwan, an average of 8 people per year are casualties of accidents involving confined spaces, proving that it is an area of concern that cannot be neglected. When misjudgments occur in confined spaces, not only can people be hurt, but they can even lose their lives, and the risks associated with confined spaces can subsequently result in rescue personnel also being killed or injured. This study was conducted via the systematic causal analysis technique (SCAT), which was proposed and developed by the International Loss Control Institute (ILCI), with the intention of identifying the critical basic causes of the confined space accidents that have occurred over the years in the Taiwan area, in order to propose corresponding improvement strategies. After investigating the statistics in Taiwan, it was determined that hydrogen sulfide was involved in 45% of accidental deaths in confined spaces, followed by 11% involving carbon dioxide, 9% involving carbon monoxide, and 7% involving toluene. Additional analysis of non-standard acts identified “failure of operating procedures” as being involved in 27% of accidents, followed by 25% involving “improper personal protective equipment” and 23% involving “incorrect position”. The analysis of non-standard conditions revealed that “dangerous workplace” was involved in 39% of accidents, “improper protective measures” in 30%, and “inadequate ventilation” in 27%. In accordance with our analysis results, it could be suggested that hazard prevention strategies for confined spaces, in addition to encouraging avoidance of non-standard acts by personnel, should also strive to improve these non-standard conditions. Otherwise, if not prevented deliberately and in a fundamental, relevant accidents will remain inevitable.

## 1. Introduction 

Serious occupational accidents caused by confined spaces have been occurring for many years. Continuous and comprehensive reports [1,2,3] and academic studies [4,5] have been published and discussed worldwide regarding confined spaces from the point of view of safety and disaster prevention. Many confined space accidents occur because workers fail to be aware of the dangers inside, and they may not consider the hazards inherent to their confined space work. Hence, the most important concern of all is the careful identification of all possible confined spaces [6]. The Occupational Safety and Health Administration (OSHA) in the United States estimates that there will be 4.8 million confined spaces created per year in America [7]. There are many potential hazards in confined spaces, primarily atmospheric hazards (e.g., poisoning, suffocation, and explosion) and physical hazards (e.g., mechanical, electrical, phagocytosis, and falling) [8]. Between 2008 and 2018 in Taiwan, an average of nearly 8 people died each year due to poisoning or suffocation in confined spaces, and, on average, 18% of occupational accidents resulted in the death of one or more people. In particular, confined space disasters often also lead to rescue casualties; an average of one person per two victims dies in attempting rescue of companions [9]. Similarly, in the United States between 1992 and 2005, an average of nearly 38 people died every year due to poisoning or suffocation in confined spaces; on average, 20% of occupational accidents caused multiple deaths [10]. In addition, in Canada between 1998 and 2011, an average of nearly three people died per year due to confined spaces, with an average of nearly 20% of occupational accidents leading to multiple deaths [11]. In a survey from Canada, indicating the types of accidents that led to people dying in confined spaces, the main cause was found to be suffocation by hydrogen sulfide in wastewater treatment plants and septic tanks [11]. Thus, the United States, Canada, and Taiwan are becoming aware of the seriousness of occupational accidents in confined spaces. In Korea, workers are killed or injured in confined spaces every year, with the victims including people working in confined spaces and those trying to rescue them without proper training and equipment [2]. To prevent accidents occurring in confined spaces, a systematic inspection should be carried out before anyone enters the confined spaces, including checking for damage of protective equipment, relevant training, management, and supervision. In other words, an employer should conduct an appropriate and adequate assessment of the risks involved in any work activities to decide which safety measures are necessary [3].

According to the law in Taiwan (“Occupational Safety and Health Facilities Regulation (Article 19-1)), a confined space is defined as a space that “is not intended inside for labor routine work, with restricted access, and inadequate natural ventilation for keeping ample and clean air” [12]. The US National Institute for Occupational Safety and Health (NIOSH) (1987) defines a confined space as “a space with limited entry/egress and inadequate ventilation, and not suitable for labor continuous work” [13]. According to another Taiwanese law, “Regulations for Prevention of Hypoxia (Article 2)”, common confined space workplaces include internal workplaces with inadequate ventilation, such as water wells, pits, vertical pits, tunnels, caissons, culverts, and manholes [14]; because “confined space” is often understood as “enclosed space”, workers may underestimate the hazards involved in entering such spaces [15]. Because confined spaces may contain toxic, hypoxic, and/or explosive gases, and may contain particular physical hazards, such as the possibility of collapse due to pipes and the contents of pipes, confined space engineering is considered to be dangerous engineering, with a high frequency of injury and lethality [4,5,10].

In previous accident investigations and relevant regulations in Taiwan, all accidents occurring in workplaces were generally defined as “occupational injury”. However, the Institute of Labor, Occupational Safety and Health in Taiwan suggests that such accidents should be classified based on the characteristics of the accidents into process accident, traffic accident, personal injury, occupational disease, or near miss [16]. One of the important purposes of occupational safety analysis of accidents is to clarify the critical causes that affected the severity of the incident [17]. In-depth safety analysis is the most important factor in ensuring safe production [18,19]. In addition, analyzing accidents based on business and injury characteristics is crucial for identifying their critical causes and implementing preventive management plans [20]. If data analysis can be applied to identify risks in an organization, it can not only increase the productivity of workers, but also reduce occupational injuries and diseases [21]. Therefore, the purpose of this study was to find the causes of higher accident frequency by means of a descriptive statistics approach using a number allocation table. A total of 64 accidents that occurred between 2008 and 2018 in confined spaces [22] due to atmospheric hazards and that caused death were further investigated via systematic causal analysis technique (SCAT) [23], in order to analyze the primary cause of catastrophic and fatal occupational accidents in confined spaces, and to provide references to business entities for the establishment of improvement strategies. 

## 2. Materials and Methods

### 2.1. Subjects 

There is rarely a single cause of occupational accidents in confined spaces; they are usually caused by multiple factors. However, cause analysis of occupational accidents in the past has often focused on unsafe actors or conditions. Nevertheless, if the cause analysis is not extended to the management system level for thorough improvement, similar accidents may happen repeatedly, potentially involving even more serious accidents. By utilizing the SCAT technique to analyze the case data of catastrophic and fatal occupational accidents associated with confined spaces from 2008 to 2018 in Taiwan, the factors that caused management system failure could be investigated in detail in addition to disclosing the main cause of accidents.

### 2.2. Data Collection 

**Figure 1:**
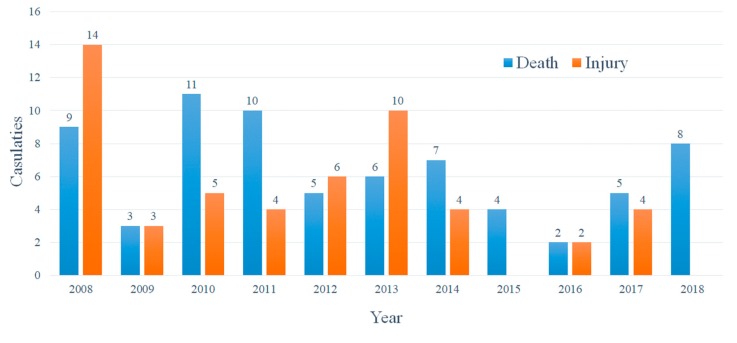
Statistics for injuries and deaths in catastrophic and fatal occupational accidents occurring in confined spaces from 2008 to 2018 in Taiwan.

Analysis of the 64 accidents reports associated with confined spaces in Taiwan from 2008 to 2018 (not limited to fatal accidents), as shown in Figure 1, revealed there were 9 deaths and 14 injuries in 2008. The Council of Labor Affairs in Taiwan, i.e., the predecessor of the Ministry of Labor Taiwan, immediately extended the number of labor inspections to include 1534 factories in 2009 and, in April of the same year, launched a “Confined Space Hazards Prevention Implementation Plan”, and 98 publicity meetings and 6 experience-sharing conferences on “Confined Space Hazards Prevention” were held [24]. In the same year, the number of deaths and injuries fell to three people in individual accidents, representing an immediate and remarkable effect. Nevertheless, in 2010, two catastrophic and fatal occupational accidents occurred involving hydrogen sulfide, resulting in seven deaths and four injuries. Although the labor inspections were increased to include 4728 factories [25], it was still impossible to recover the lost life.

**Figure 2:**
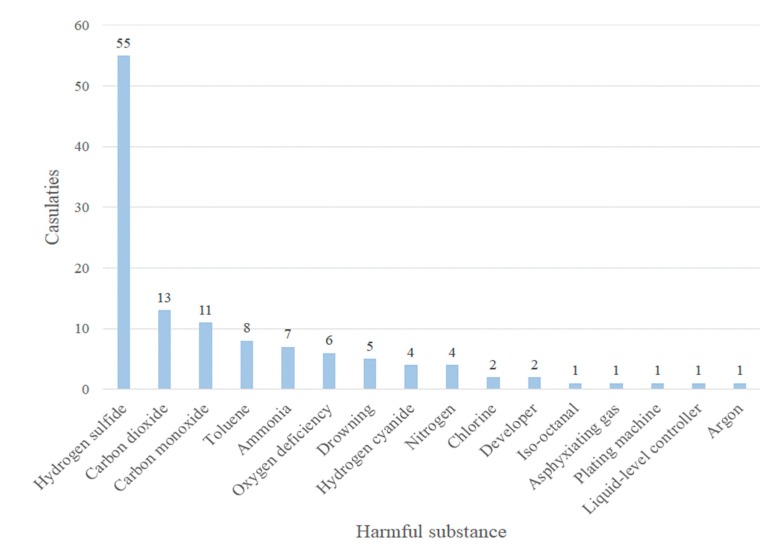
Classification statistics for media and casualties of confined space accidents in Taiwan from 2008 to 2018.

A total 64 accident reports associated with confined spaces were consolidated, as shown in Table 1; in total, 70 deaths and 52 injuries were included. Further analysis, as illustrated in Figure 2, indicated that the harmful substance associated with the highest proportion of casualties was hydrogen sulfide with 45% (55/122), followed by carbon dioxide with 11% (13/122), carbon monoxide with 9% (11/122), toluene with 7% (8/122), and ammonia with 6% (6/122). Therefore, if these five hazards could be eliminated, the proportion of disasters could be reduced.

### 2.3. Data Analysis

**Figure 3:**
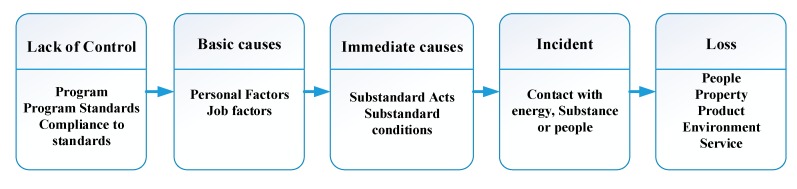
The ILCI loss causation model.

In 1931, Heinrich proposed the “domino theory”, which divides the causes of accidents into five factors: social factors, personal negligence, unsafe acts, accidents, and injuries; these factors will affect each other. Each accident factor is considered a domino. When the initial event occurs, other accident factors will also take place sequentially. The logical and general principle of the domino theory is that if one or more dominoes are removed, the whole row of dominoes will not fall down [26,27]. In particular, if the last two dominoes are removed, accidents and injuries will not happen. Most accident investigations conducted by business entities in Taiwan identified and determined risk factors in accordance with this theory and the responsibilities of the law, but were often limited to discussions of human and device factors, so the root causes of accidents could not be confirmed. Therefore, this study was based on the ILCI loss causation model [28], which was developed by the International Loss Control Institute (ILCI), as displayed in Figure 3. Combined with the SCAT check list of Pei-Ling Yao based on Bird and Germain, the direct cause of each accident was divided into 16 items related to non-standard acts and 13 items related to non-standard conditions [29], as delineated in Table 2. According to the interpretation of direct cause based on CCPS, the direct cause is the factor that directly causes an incident, with no other factor’s intervention, and is also the closest cause to the time and space of the accident [30]. Therefore, in this study, the catastrophic and fatal occupational accidents associated with confined spaces in Taiwan from 2008 to 2018 were discussed using SCAT in order to clarify their direct causes and to recognize hazards or identify the cause of failure in risk control methods, in order to develop corresponding improvements and preventive measures.

**Figure 4:**
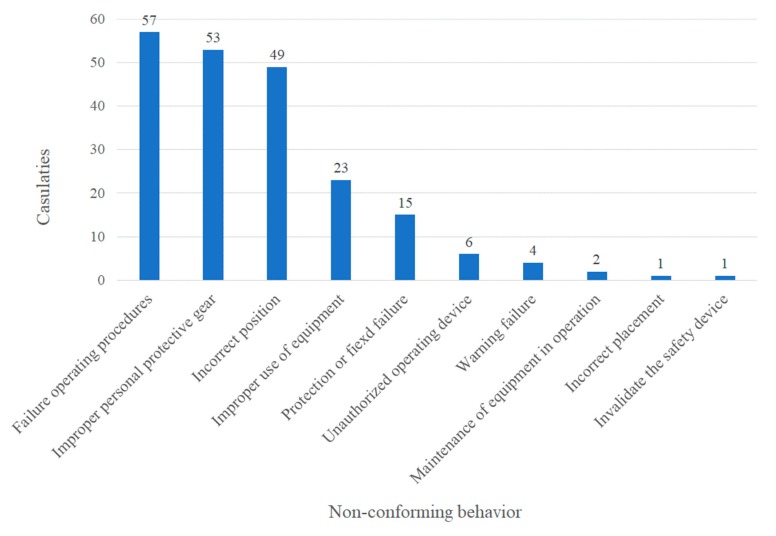
Statistics for non-standard acts.

**Figure 5:**
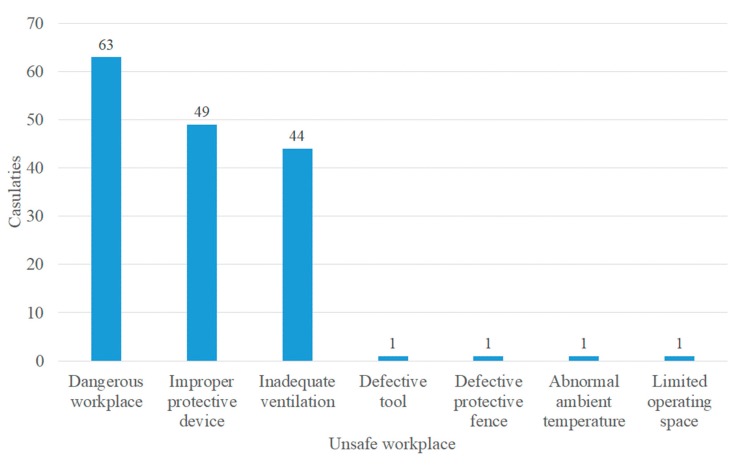
Statistics for non-standard conditions.

The analysis of non-standard acts conducted via SCAT is displayed in Figure 4, indicating that the highest proportion of direct causes was “failure of operating procedures” with 27% (57/211), followed by “improper personal protective equipment” with 25% (53/211) and incorrect position” with 23% (49/211). In addition, the analysis of non-standard conditions displayed in Figure 5 revealed that “dangerous workplace” had the highest proportion of direct causes with 39% (63/160), followed by “improper protective measures” with 30% (49/160) and “inadequate ventilation” with 27% (44/160).

## 3. Results and Discussion 

### 3.1. Characterization of Fatal Work Accidents in Confined Spaces in Taiwan

In this study, consolidation analysis was conducted according to the 64 accident reports associated with confined spaces published by OSHA (Taiwan). Relevant analysis results were as follows.

#### 3.1.1. Period of the Year

There were 11 catastrophic and fatal occupational accidents associated with confined spaces in 2008 in Taiwan, with a total of 9 deaths and 14 injuries. These casualties accounted for 19% (23/122) of the casualties recorded in the statistical period, and contributed to the passing of the “Occupational Safety and Health Promotion Program” by Taiwan Executive Yuan Taiwan in 2009, which involved eradication of inspection-oriented thinking, adoption of the three-in-one inspection strategy of “promotion, inspection, counseling” to promote the risk management grading inspection system, and establishment of the “Declaration System for Confined Space Work Schedule”. Moreover, the publicity meetings were greatly increased to a total 98; additionally, six experience-sharing conferences on “Confined Space Hazards Prevention” were held [31]. The number of casualties in 2009 was significantly reduced to six people. This plan continued to be implemented until 2010; since then, the number of casualties in confined space accidents has continued to decline steadily.

#### 3.1.2. Type of Accident

Table 3 gives a detailed description of the harmful substances involved in casualties caused in confined spaces investigated in Taiwan; hydrogen sulfide was the main harmful substance involved in accidents, and these accidents made up 45% (55/122) of casualties. Among these, 19 deaths occurred in wastewater treatment facilities, 3 deaths occurred in sewers, and 2 deaths occurred in hot spring storage tanks. Therefore, the number of casualties due to hydrogen sulfide accidents accounted for half of the total. Analysis of the types of non-standard acts according to SCAT, as shown in Table 4, indicated that “failure of operating procedures” was the cause of the highest proportion of accidents at 27% (57/211), followed by 25% (53/211) due to “improper personal protective equipment” and 23% (49/211) due to “incorrect position”. From further cross-comparison between “hydrogen sulfide” and “non-standard acts”, we found that the cause of “incorrect position” accounted for 43% (21/49) of casualties, “improper personal protective equipment” for 42% (22/53), and “failure of operating procedures” for 40% (23/57). From the analysis of the types of non-standard conditions, as shown in Table 5, the cause of “dangerous workplace” was associated with the highest proportion of accidents at 39% (63/160), followed by “improper protective measures” with 30% (49/160), and “inadequate ventilation” with 27% (44/160). From further cross-comparison between “hydrogen sulfide” and “non-standard conditions”, it was found that the cause of “inadequate ventilation” accounted for 45% (20/44) of casualties, “improper protective measures” for 35% (17/49), and “dangerous workplace” for 33% (21/63).

According to the analysis of the direct causes made via SCAT, the first three causes of “non-standard acts” were associated with 75% of the total accidents; the first three causes of “non-standard conditions” were associated with 96%; if the appropriate coping strategy (as delineated in Table 3) is applied to address these causes, the probability of confined space accidents due to hydrogen sulfide should be reduced.

The results revealed that, due to the lack of awareness of the confined space hazards by employers, problems arise for personnel, such as lacking in education and training relevant for the work, or being immersed in a suffocating or anoxic workplace without knowing, and therefore being unable to provide appropriate personal protective equipment and adequate ventilation measures. In addition, rescue people often underestimated the risk involved in entering the accident sites, thus causing themselves to be killed or injured subsequently.

The suggested strategies for preventing accidents represent the most important part of the results and conclusions of this study. First, through our research outcomes, we found that the hydrogen sulfide was the most common harmful substance involved in accidents in confined spaces, involved in 45% of accidents. Therefore, in order of priority, we began with hydrogen-sulfide-related strategies, as these were the most common accidents (up to almost 50%), when listing suggested coping strategies and practical considerations for preventing confined space accidents, as displayed in Table 6. We paid more attention and space to coping strategies for factors that were the causes of greater numbers of accidents. Table 7 presents the suggested coping strategies for preventing confined space accidents caused by carbon dioxide. Thus, we suggested coping strategies for the causes of more than half of confined space accidents i.e., hydrogen sulfide 45% (Table 6) and carbon dioxide 11% (Table 7), meaning this study could be a practical reference for confined space hazard prevention.

From the point of view of preventing confined space accidents, coping strategies have been suggested for the causes of than half of all confined space accidents (hydrogen sulfide and carbon dioxide), and are included in Table 6 and Table 7, respectively.

## 4. Conclusions 

Referring to the 64 accident reports and 70 death cases analyzed in this study, we were able to more accurately identify the factors that cause confined space hazards. An average of 18% of the confined space occupational accidents that occur in Taiwan cause multiple deaths [5]. In addition, in Canada, nearly 20% of confined space occupational accidents have led to multiple deaths [6]. Since the threats of confined space hazards are typically silent, they often result in catastrophic and fatal occupational accidents with multiple deaths. 

This study analyzed the causes of confined space occupational disasters via an improved SCAT check method. The original SCAT checked method does not specify the definition of the direct cause. This would make it difficult for investigators to determine the true cause of an accident. Therefore, this study supplied a judgment of relevant direct causes, and also provided a benchmark that could be used to determine the multiple causes of an accident in detail, and could assist investigators to quickly, effectively, and objectively determine the true cause of an accident. In our analysis of confined space disasters, whether in Taiwan or Canada, suffocation by hydrogen sulfide was found to be the main cause of casualties. After cross-comparison of hydrogen sulfide with non-standard acts and non-standard conditions, it was found that the causes of “incorrect position” and “inadequate ventilation” were associated with the highest proportion of accidents. The research’s limitations and implications, their implications, expected contributions, and our future prospects are delineated below. This study used SCAT as a preliminary analysis method for confined space accidents, and did not further analyze indirect causes on top of direct causes. In the future, case studies could be used to continue in-depth research. After classifying the evidence data using the OSHA 4Ps model, the ECFA + method was used to construct a time series diagram, and the SCAT accident cause checklist was used to design related question forms to assist investigators to ascertain the direct, indirect and root causes of accidents. According to Table 6 and Table 7, preventive coping strategies for eliminating the hazards of confined space work should start by removing non-standard acts and non-standard conditions, and should especially focus on design for intrinsic safety, so as to minimize confined space hazards to nonexistence. 

A confined space is a work area wherein mistakes cannot be accepted. When problems occur in confined spaces, people not only get hurt but may even lose their lives. Nevertheless, there are still many employers who do not comply with the relevant laws and regulations, exposing their workers to hazardous situations. Such employers may not understand the real risks, and may make their workers enter work spaces with insufficient protection. Such situations are quite dangerous and have caused workers to be killed. Never wait for the labor inspection agency to find the problem, and do not wait until the emergency occurs to respond to the problem. Be sure that hazard recognition and identification are done well in advance, find problems at any time, and then make corrections on a continuous basis.

## Figures and Tables

**Figure 6:**
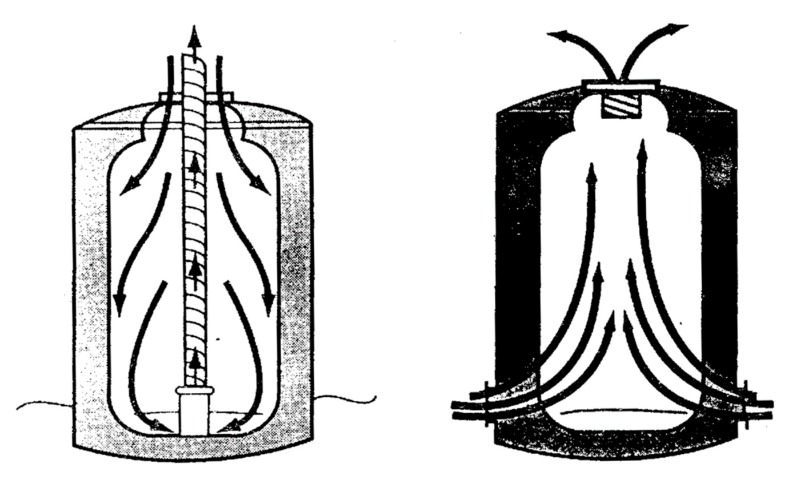
Schematic diagram of a ventilation method for a heavier-than-air hazardous gas (left) and a lighter-than-air hazardous gas (right).

**Table 1:** Statistics for catastrophic and fatal occupational accidents associated with confined spaces in Taiwan from 2008 to 2018.

Business	Medium	Accident Type	Time of Occurrence	Death	Injury
Interior decoration and repair	Harmful substance (toluene)	Contact with harmful substance	2008		2
Mechanical and electrical maintenance	Harmful substance (carbon monoxide)	Contact with harmful substance	2008	2	
Interior decoration and repair	Harmful substance (carbon monoxide)	Contact with harmful substance	2008		3
Interior decoration and repair	Harmful substance (toluene)	Contact with harmful substance	2008		2
Wholesale, repair, and installation construction of firefighting equipment	Harmful substance (carbon dioxide)	Contact with harmful substance	2008		6
Environmental protection engineering	Other (nitrogen)	Contact with harmful substance	2008	1	
Manufacture of other chemical products	Harmful substance (hydrogen sulfide)	Contact with harmful substance	2008		1
Casting of iron and steel	Harmful substance (carbon monoxide)	Contact with harmful substance	2008	1	
Raising of swine/pigs	Harmful substance (carbon monoxide, etc.)	Contact with harmful substance	2008	1	
Mechanics, telecommunications, and electrical engineering, security service activities	Harmful substance (carbon monoxide, etc.)	Contact with harmful substance	2008	2	
Building maintenance and upholstery	Harmful substance (toluene)	Contact with harmful substance	2008	2	
Wastewater and sewage treatment	Harmful substance (hydrogen sulfide)	Contact with harmful substance	2009		3
Wholesale of electrical equipment	Harmful substance (developer)	Contact with harmful substance	2009	2	
Manufacture of semiconductors	Other (nitrogen)	Contact with harmful substance	2009	1	
Electrical equipment	Harmful substance (ammonia)	Contact with harmful substance	2010	1	
Pipeline engineering	Harmful substance (hydrogen sulfide)	Contact with harmful substance	2010	1	
Processing of fish, crustaceans, and mollusks	Harmful substance (hydrogen sulfide)	Contact with harmful substance	2010	2	4
Manufacture of other leather and fur products	Harmful substance (hydrogen sulfide)	Contact with harmful substance	2010	5	
Other special-purpose machinery manufacturing and repair	Harmful substance (hydrogen sulfide)	Contact with harmful substance	2010	1	1
Casting of iron and steel	Harmful substance (carbon monoxide)	Contact with harmful substance	2010	1	
Manufacture of other rubber products	Harmful substance (hydrogen sulfide)	Contact with harmful substance	2011	1	1
Manufacture of basic chemicals	Other (nitrogen)	Contact with harmful substance	2011	1	
Manufacture of basic chemicals	Other (nitrogen)	Contact with harmful substance	2011	1	
Manufacture of other chemical products	Harmful substance (ammonia)	Contact with harmful substance	2011		1
Slaughtering	Harmful substance (Hydrogen sulfide)	Contact with harmful substance	2011	1	1
Manufacture of bare printed circuit boards	Other (plating machine)	Drowning	2011	1	
Raising of swine/pigs	Harmful substance (hydrogen sulfide)	Contact with harmful substance, etc.	2011	1	
Environment technical testing	Water	Drowning	2011	1	
Treatment of metal surface	Harmful substance (hydrogen cyanide)	Contact with harmful substance, etc.	2011	3	1
Other (manufacture of fertilizers)	Harmful substance (carbon dioxide)	Contact with harmful substance	2012	1	
Interior decoration and repair	Harmful substance (toluene)	Contact with harmful substance	2012		2
Warehousing and storage	Harmful substance (carbon dioxide)	Contact with harmful substance	2012	1	
Food industry	Harmful substance (carbon monoxide)	Contact with harmful substance	2012		1
Wastewater (sewage) treatment	Harmful substance (hydrogen sulfide)	Contact with harmful substance	2012	1	
Pipeline construction	Harmful substance (hydrogen sulfide)	Contact with harmful substance	2012		3
Treatment of metal surface	Water	Drowning	2012	1	
Wastewater (sewage) treatment	Water	Drowning	2012	1	
Manufacture of screws, nuts, and rivets	Harmful substance (argon)	Contact with harmful substance	2013	1	
Manufacture of petroleum and coal products	Harmful substance (hydrogen sulfide)	Contact with harmful substance	2013	1	3
Treatment of metal surface	Harmful substance (chlorine)	Contact with harmful substance	2013		2
Other specialized construction activities	Other (oxygen deficiency)	Contact with harmful substance	2013	2	
Wastewater (sewage) treatment	Harmful substance (hydrogen sulfide)	Contact with harmful substance	2013		2
Manufacture of other non-metallic mineral products	Water	Drowning	2013	1	
Manufacture of beer	Harmful substance (carbon dioxide)	Contact with harmful substance, etc.	2013		3
Manufacture of beer	Other (oxygen deficiency)	Contact with harmful substance, etc.	2013	1	
Manufacture of paper	Water	Drowning	2014	1	
Petrochemical manufacturing	Harmful substance (iso-octanol, etc.)	Contact with harmful substance, etc.	2014	1	
Wastewater (sewage) treatment	Harmful substance (hydrogen sulfide)	Contact with harmful substance, etc.	2014	1	
Manufacture of other chemical products	Other (oxygen deficiency)	Contact with harmful substance, etc.	2014	1	
Fire protection engineering	Harmful substance (carbon dioxide)	Contact with harmful substance, etc.	2014	1	1
Construction of other civil engineering projects	Harmful substance (hydrogen sulfide)	Contact with harmful substance	2014	1	1
Sewage work	Harmful substance (ammonia)	Contact with harmful substance	2014	1	2
Renting and leasing of agricultural and other industrial machinery and equipment	Electric transmission and distribution wire (liquid-level controller control wire)	Electric shock	2015	1	
Construction	Harmful substance (hydrogen sulfide)	Contact with harmful substance	2015	2	
Wastewater (sewage) treatment	Harmful substance (hydrogen sulfide)	Contact with harmful substance	2015	1	
Mechanics, telecommunications, and electrical facilities installation	Other (oxygen deficiency)	Contact with harmful substance, etc.	2016	1	1
Hot spring industry	Harmful substance (hydrogen sulfide)	Contact with harmful substance	2016		1
Plumbing, heat, and air-conditioning installation	Other (asphyxiating gas)	Contact with harmful substance, etc.	2016	1	
Manufacture of electronics	Harmful substance (hydrogen sulfide)	Contact with harmful substance	2017	4	2
Plumbing, heat, and air-conditioning installation	Harmful substance (hydrogen sulfide)	Contact with harmful substance	2017	1	2
Plumbing, heat, and air-conditioning installation	Harmful substance (hydrogen sulfide)	Contact with harmful substance	2018	1	
Wastewater (sewage) treatment	Harmful substance (hydrogen sulfide)	Contact with harmful substance	2018	2	
Retail sale of construction materials in specialized stores	Harmful substance (hydrogen sulfide)	Contact with harmful substance	2018	3	
Repair and installation of machinery and equipment	Harmful substance (ammonia)	Contact with harmful substance	2018	2	

**Table 2:** Checklist for direct causes defined using SCAT.

Non-Standard Act	Non-Standard Condition
1. Unauthorized operation of equipment 2. Warning failure 3. Protection or fixed failure 4. Not operating at the regulation speed 5. Invalidation of the safety device 6. Use of defective equipment 7. Improper personal protective equipment 8. Inappropriate or incorrect loading 9. Inappropriate or incorrect placement 10. Inappropriate or incorrect handling 11. Incorrect position 12. Inappropriate maintenance of equipment in operation 13. Horseplay 14. Personnel affected by alcohol or drugs 15. Improper use of equipment 16. Failure of operating procedures	17. Inappropriate protective cover or fence 18. Improper protective measures 19. Defective tool or equipment 20. Crowded environment or restricted action 21. Inappropriate warning system 22. Fire and explosion hazard 23. Internal poor rectification/disorder 24. Noise exposure 25. Radiation exposure 26. Extreme temperature 27. Poor lighting 28. Inadequate ventilation 29. Dangerous workplace

**Table 3:** Harmful substances involved in casualties caused by confined space examined in this study.

Harmful Substance	Explosion Limits (%)	Casualties
Hydrogen sulfide	4.0–4.4	55
Carbon dioxide	－	13
Carbon monoxide	－	11
Toluene	1.2–7.1	8
Ammonia	15.0–28.0	7
Oxygen deficiency	－	6
Drowning	－	5
Hydrogen cyanide	5.6–40.0	4
Nitrogen	－	4
Chlorine	－	2
Developer	－	2
Iso-octanal	1.0–6.0	1
Asphyxiating gas	－	1
Plating machine	－	1
Liquid-level controller	－	1
Argon	－	1

**Table 4:** Non-standard acts and non-standard conditions analyzed in this study.

Non-Standard Act	Number of Times	Non-Standard Condition	Number of Incidents
Failure of operating procedures	57	Dangerous workplace	63
Improper personal protective equipment	53	Improper protective measures	49
Incorrect position	49	Inadequate ventilation	44
Improper use of equipment	23	Defective tool	1
Protection or fixed failure	15	Defective protective fence	1
Unauthorized operation of equipment	6	Abnormal ambient temperature	1
Warning failure	4	Limited operating space	1
Inappropriate maintenance of equipment in operation	2		0
Incorrect placement	1		0
Invalidation of the safety device	1		0

**Table 5:** Key causes of exposure to harmful substances by non-standard acts and non-standard conditions analyzed in this study.

Harmful Substance	Non-Standard Act	Non-Standard Condition
Hydrogen sulfide	1. Incorrect position (21/49) 2. Improper personal protective equipment (22/53) 3. Failure of operating procedures (23/57)	1. Inadequate ventilation (20/44) 2. Improper protective measures (17/49) 3. Dangerous workplace (21/63)
Carbon dioxide	1. Improper personal protective equipment (13/53) 2. Incorrect position (7/49) 3. Failure of operating procedures (7/57)	1. Inadequate ventilation (13/44) 2. Dangerous workplace (13/63) 3. Improper protective measures (9/49)
Carbon monoxide	1. Incorrect position (11/49) 2. Failure of operating procedures (11/57) 3. Improper personal protective equipment (7/53)	1. Inadequate ventilation (9/44) 2. Dangerous workplace (11/63) 3. Improper protective measures (7/49)

**Table 6:** Coping strategies for preventing confined space accidents due to hydrogen sulfide.

Direct Cause	Description	Coping Strategy
Non-standard act	Incorrect position	1. The entrance to the space could be made small enough to make it impossible for a worker to pass through. 2. If an available opening or channel is large enough for a worker to pass through, it should be closed. 3. Observation holes and cleaning openings should be installed in tanks and other equipment, so that workers can view and clean the interior without entering the space.
	Improper personal protective equipment	1. When working in hypoxic conditions, make sure workers wear respiratory protection, such as air respirators, and prepare ladders, safety belts, or life lines. 2. For related personnel (including rescue people), implement training in first aid for H_2_S exposure, and conduct evacuation training. 3. Ensure that the tools entering the confined space do not cause hazards.
	Failure of operating procedures	1. Carry out safety and health education and training on confined spaces. 2. Cultivate the capability of hazard recognition and identification for confined space work. 3. Set the appropriate SOP (standard operating procedure).
Non-standard condition	Inadequate ventilation	1. Install mechanical ventilation with continuous operation or a door-controlled switch, to control air quality in confined spaces. 2. Implement ventilation and oxygen concentration measurement before operation, and set up an alarm to be raised when ventilation is ineffective. In addition, consider installing stationary gas equipment with alarms to monitor air quality. 3. If dangerous gases are present, ventilation channels should be provided at regular intervals and multiple openings should be installed at opposite ends of the space for complete and effective ventilation.
	Improper protective measures	1. Install standard steps with handrails instead of climbing stairs or spiral stairs. 2. Install critical equipment that must be regularly operated, inspected, or maintained (e.g., valves, meters, etc.) outside the space, so that it is unnecessary to enter inside. 3. Ensure that all electrical equipment is properly enclosed, grounded, and certified for using in specific environments.
	Dangerous workplace	1. Set up oxygen and harmful gas (H_2_S, CH_4_) detection systems to monitor the internal conditions of the working environment. 2. Ensure that all machinery and equipment is properly protected and that all electrical equipment is properly sealed. 3. Choose the machinery and equipment with the longest service life and the lowest maintenance requirements to reduce the number of times the confined spaces must be entered.

**Table 7:** Coping strategies for preventing confined space accidents due to carbon dioxide.

Direct Cause	Description	Coping Strategy
Non-standard act	Improper personal protective equipment	1. When working in hypoxic conditions, make sure workers use personal protective equipment, such as air respirators, supplied air respirators (SAR), etc., or emergency aids, tripods, double-hook backpack-type safety belt, etc. 2. For related personnel (including rescue people), implement training on first aid for CO_2_ inhalation and evacuation training. 3. Ensure that tools entering the confined space do not cause hazards.
	Incorrect position	1. The space entrance should be made small enough to make it impossible for a worker to pass through. 2. If an available opening or channel is large enough for a worker to pass through, it should be closed. 3. Install observation holes and cleaning openings in tanks and other equipment, so that workers can view and clean the interior without entering the space.
	Failure of operating procedures	1. Carry out safety and health education and training on confined spaces. 2. Cultivate the capability of hazard recognition and identification for confined space work. 3. Set the appropriate SOP (standard operating procedure).
Non-standard condition	Inadequate ventilation	1. Install mechanical ventilation with continuous operation or door-controlled switches to control air quality in confined spaces. 2. Implement ventilation and oxygen concentration measurement before operation, and set up an alarm to be raised when ventilation is ineffective. In addition, consider installing stationary gas equipment with alarms to monitor air quality. 3. Carbon dioxide is a heavier-than-air hazardous gas, and its ventilation method should be paid attention to. (The left part of Figure 6 shows a schematic diagram of a ventilation method for heavier-than-air hazardous gases.)
	Dangerous workplace	1. Measure the concentration of oxygen and hydrogen sulfide in the place, and choose three or more points in the horizontal and vertical directions. If you cannot confirm that the oxygen concentration is above 18%, never let workers enter. 2. Workers should enter only after inhaling 5 times the volume of harmless air in the place. During operation, pay attention to the uniformity of ventilation, and continuously supply air at a rate of 20 times per hour.
	Improper protective measures	1. Install standard steps with handrails instead of climbing stairs or spiral stairs. 2. Install the critical equipment that must be regularly operated, inspected, or maintained (e.g., valves, meters, etc.) outside the space, so that it is unnecessary to enter inside.
